# Glycosaminoglycans Metabolism

**DOI:** 10.1155/2012/245792

**Published:** 2012-04-24

**Authors:** Manuela Viola, Timothy E. L. Douglas, Laura Alaniz, Barbara Bartolini

**Affiliations:** ^1^Department of Surgical and Morphological Sciences, University of Insubria, via J.H. Dunant 5, 21100 Varese, Italy; ^2^Department of Biomaterials, Radboud University Medical Center Nijmegen, 6500 HB Nijmegen, The Netherlands; ^3^Polymer Chemistry and Biomaterials (PBM) Group, Department of Organic Chemistry, Ghent University, Krijgslaan 281 S4, 9000 Ghent, Belgium; ^4^Gene Therapy Laboratory, Liver Unit, School of Biomedical Sciences, Austral University, CONICET, B1629AHJ Pilar, Buenos Aires, Argentina; ^5^Department of Experimental Medical Science, Biomedical Center D12, Lund University, Tornavägen 10, SE-221 84 Lund, Sweden

Glycosaminoglycans (GAGs) are unbranched, polysaccharide chains which, with the exception of hyaluronan (HA), are highly sulphated and constitute the glucidic moieties of proteoglycans (PGs) macromolecules. Depending on monosaccharide composition and protein linkage region, as well as sulphation pattern and degree, GAGs can be grouped in four subfamilies named chondroitin/dermatan sulphate (CS/DS), heparan sulphate/heparin (HS/HE), hyaluronan (HA), and keratan sulphate (KS). 

GAGs contribute to the PGs native folding and functions, as well as to tissue and organ behavior. In fact, GAGs are involved in stabilization of the fibrillar extracellular matrix (ECM), control of hydration, regulation of tissue, and organism development by controlling cell cycle, cell behavior, and differentiation. HA synthesis is a process carried out on plasma membrane by specific enzymes called Hyaluronan synthases. The other GAGs chains are polymerized in the ER and Golgi compartments by specific enzymes drawn close in a complex named GAGosome. The modification of the synthesis of the GAG portion of PGs or HA can alter significantly the ECM structure and composition, with multiple effects, leading to physiological events such as tissue ageing and pregnancy or pathological events: kidney agenesis, cardiac malformations, abnormal mast cells, somatic overgrowth, lung dysfunction, chondrodysplasia, tumor progression, and fibrosis process.

On the other hand, the same studies about GAGs involvement in cell differentiation have led in the last decade to the use of GAG chains in the preparation of innovative and biocompatible materials. GAGs are incorporated into polymer scaffolds for tissue regeneration in order to improve their physiochemical properties, such as water-binding ability, and to influence cell behavior. Certain biomaterials contain GAGs as their main component. For example, in biomaterials aimed to blood contact, heparin is applied to counteract blood clotting. Furthermore, growth factors are often applied together with scaffolds and GAGs, due to the well known ability of various GAGs to bind specific growth factors and to modulate their activity and bioavailability.

In this special issue on GAGs, we have invited papers that address such issues, both by original work and reviews.

One of the papers of this special issue shows that CS/DS GAGs and HA are differently distributed between pericardium and valves and within heart valves themselves before and after decellularization. Distribution of glycosaminoglycans is also dependent from the vascular district and topographic localization. Data presented suggest that both decellularized porcine heart valves and bovine pericardium represent a promising material for future development of tissue engineered heart valve scaffolds.

The paper by E. Zinellu et al. compares the amount and sulphation characteristic of CS present in plasma of patients with hard and soft carotid arteriosclerotic plaques to the one found in plasma of healthy volunteers. The authors suggest that the differences evidenced in the paper could be used to develop new diagnostic tests for atherosclerosis.

The authors of the paper entitled “*Glycosaminoglycan storage disorders: a review*” reviewed the clinical consequences of alteration in degradation of GAGs. Intralysosomal accumulation of undegraded products causes a group of lysosomal storage disorders known as mucopolysaccharidoses (MPSs). They provide an overview of the molecular basis, enzymatic defects, clinical manifestations and diagnosis of each MPSs, focusing also on the available animal models and describing potential perspectives of therapy for each one.

Other authors documented the effects on diabetic nephropathy of the treatment with low-molecular-weight heparin (dalteparin, LMW-HE). Patients with type 2 diabetes and with neuro-ischemic foot ulcers were given LMW-HE for a maximum of six months. The authors showed that LMW-HE is beneficial for the outcome of neuro-ischemic foot ulcers and it has no effect on glomerular function despite an increment of excretion of GAGs. Moreover they stated that urinary IgM and IgG (consequence of alterations of the size-selective properties of the glomerular capillary wall) seem to be better markers than albuminuria for detecting and predicting renal injury in the patients. Evidences from this paper contribute to highlight the complex role of the GAG heparin, which can influence directly and indirectly the structure and the function of the glomerular capillary wall and therefore plays a pivotal role in diabetes and kidney diseases.

One of the papers investigates more on the same topic, that is, the importance of GAGs distribution on the endothelial surface, by focusing on a different pathology. Starting from defining the problem of acute coronary syndrome, the authors described the importance of a good reperfusion therapy on the recovery of a proper microcirculation in damaged heart tissue. An adequate capillary circulation is a necessary prerequisite for normal perfusion and organ functioning, and a no-reflow phenomenon is defined as incomplete reperfusion at the microcirculatory level. Under this perspective, the composition of the endothelial glycocalyx in terms of GAGs, PGs, and glycoproteins is significant in defining its role as protective layer of the vascular wall. The no-reflow phenomenon is therefore strictly related to the composition of the glycocalyx, and the authors discuss how the deep knowledge of this layer (drug targeting, ultrastructure, and degradation) could be helpful in the best reperfusion outcome.

Another paper reviews the occurrences, the synthesis, and the catabolism of hyaluronan, followed by a description of HA receptors and the relevance of the chain length in affecting different aspects of cell and tissue behavior. The authors also mention the role of hyaluronan in malignancy and skin, discussing its new recently discovered functions.

In the same field, the paper by M. A. Solis et al. of the issue combines the review of hyaluronan properties and biological activities (strictly dependent on its dimension and receptors) with the research for a perfect microenvironment in which stem cells can be grown and/or differentiated. HA has a pivotal role in development and differentiation of cells, tissue, and organism, and stem cells present most of the HA receptor on their membrane, indicating the perfect match for their appropriate niche biomaterial.

*Manuela Viola* *Timothy E. L. Douglas* *Laura Alaniz* *Barbara Bartolini*

